# Predictive value of peripheral blood biomarkers in patients with non-small-cell lung cancer responding to anti-PD-1-based treatment

**DOI:** 10.1007/s00262-023-03620-2

**Published:** 2024-01-17

**Authors:** Shu Su, Fungjun Chen, Xin Lv, Liang Qi, Zhou Ding, Wei Ren, Ming Wei, Ye Liu, Lixia Yu, Baorui Liu, Lifeng Wang

**Affiliations:** 1 The Comprehensive Cancer Center of Nanjing Drum Tower Hospital, Affiliated Hospital of Medical School of Nanjing University & Clinical Cancer Institute of Nanjing University, Nanjing, 210032 Jiangsu China; 2 Nanjing Drum Tower Hospital Clinical College of Nanjing University of Chinese Medicine, Nanjing, China

**Keywords:** Biomarker, NSCLC, PD-1

## Abstract

**Background:**

The introduction of the anti-PD-1 antibody has greatly improved the clinical outcomes of patients with non-small cell lung cancer (NSCLC). In this study, we retrospectively analyzed the efficacy of PD-1 antibody-based therapy in patients with locally advanced inoperable or metastatic NSCLC and reported an association between peripheral blood biomarkers and clinical response in these patients.

**Methods:**

This single-center study included medical record data of patients with NSCLC treated with the PD-1 antibody as a first-line or subsequent line of treatment, either as monotherapy or in combination with chemotherapy. The patients were enrolled from 2020 to 2022. We dynamically evaluated multiple Th1 and Th2 cytokines in the blood serum and analyzed the phenotype of T cells from the peripheral blood to explore the correlation between cytokine levels, T cell phenotypes, and clinical response.

**Results:**

A total of 88 patients with stage IIIA-IV NSCLC were enrolled, out of which 60 (68.18%) achieved a partial response (PR), 13 (14.77%) had stable disease (SD), and 15 (17.05%) experienced disease progression (PD). The disease control rate was 82.95%. Our results suggested a significant reduction (*P* = 0.002, *P* < 0.005) in lymphocyte absolute counts after treatment in patients with PD. Higher levels of IFN-*γ* (*P* = 0.023, *P* < 0.05), TNF-*α* (*P* = 0.00098, *P* < 0.005), IL-4 (*P* = 0.0031, *P* < 0.005), IL-5 (*P* = 0.0015, *P* < 0.005), and IL-10 (*P* = 0.036, *P* < 0.05) were detected in the peripheral blood before treatment in the PR group compared to the PD group. Moreover, patients with high levels of IL-5, IL-13, IL-4, IL-6, IFN-*γ*, and TNF-*α* (> 10 ng/mL) had superior progression-free survival compared to those with low levels (< 10 ng/mL). Furthermore, PD-1 expression on CD8^+^ T cells was higher in patients who showed a PR than in those who did not show a response (SD + PD; *P* = 0.042, *P* < 0.05).

**Conclusions:**

The findings of this study imply that the decrease in absolute blood lymphocyte counts after treatment is correlated with disease progression. Serum cytokine levels may predict the effectiveness and survival rates of anti-PD-1 blockade therapy in patients with NSCLC. In addition, PD-1 expression on CD8^+^ T cells was positively associated with better clinical response. Our findings highlight the potential of peripheral blood biomarkers to predict the effectiveness of PD-1-targeted treatments in patients with NSCLC. Larger prospective studies are warranted to further clarify the value of these biomarkers.

## Introduction

Lung cancer is a major cause of mortality, accounting for approximately 1.6 million deaths annually worldwide [1, 2]. Clinical trials have demonstrated that the administration of anti-PD-1 or PD-L1 antibodies to patients with advanced NSCLC has shown beneficial clinical outcomes, such as improved clinical response and survival rates. Non-small-cell lung cancer (NSCLC), which accounts for 80–85% of the cases, and small-cell lung cancer (SCLC) are the two major subtypes of lung cancer. In less than a decade, immune checkpoint inhibitors have become the standard treatment for NSCLC. Nevertheless, recent clinical trials involving anti-PD-1 therapy have revealed that only 15–25% of patients with NSCLC responded to immune checkpoint blockade therapy alone, regardless of their PD-L1 expression [3–5]. Predictive biomarkers may help to identify patients with lung cancer who may benefit from PD-1/PD-L1 immunotherapy. Numerous studies have certified PD-L1 (programmed death-ligand 1) and TMB (tumor mutational burden) as the most reliable biomarkers [6]. However, to date, PD-L1 expression is the only validated predictive factor for NSCLC [7]. This has some limitations. For instance, some patients respond to PD-1/PD-L1 immunotherapy even in the absence of PD-L1 expression. Traditional therapies, such as chemotherapy, radiotherapy, or targeted therapy, can significantly affect the expression pattern of PD-L1. As a matter of fact in most clinical situations, most of the patients had received chemoimmunotherapy instead of mono-immunotherapy regardless of PD-L1 expression. The KEYNOTE-407 study showed that first-line treatment with pembrolizumab combined with chemotherapy can significantly prolong OS and PFS in metastatic squamous cell carcinoma and all populations with PD-L1 levels. The 4-year follow-up results of the KEYNOTE-189 study also showed that regardless of PD-L1 expression levels, the combination of pembrolizumab and chemotherapy can bring survival benefits in adenocarcinoma of NSCLC. Other predictive biomarkers including TMB and gene expression profiling are still under investigation; however, these tumor-associated markers are costly and invasive. Hence, there is an urgent need for peripheral blood biomarkers to help identify responding patients with NSCLC in clinical situations, as these biomarkers would be more cost-effective and clinically convenient.

Previous studies have proposed that a number of peripheral blood markers could be indicative of PD-1/PD-L1 inhibition in lung cancer, such as the neutrophil–lymphocyte ratio (NLR); platelet-lymphocyte ratio (PLR); neutrophil count [8], absolute monocyte count (AMC); absolute eosinophil count (AEC); and serum biomarkers such as lactate dehydrogenase (LDH), plasma-albumin (ALB), and C-reactive protein (CRP)[9]. PD-1 or PD-L1 expression on certain immune cell populations has been identified as biomarkers in response to immunotherapy in lung cancer. Moreover, the T-cell receptor (TCR) repertoire diversity of peripheral PD-1^+^ T cells is thought to predict clinical outcomes after immunotherapy in patients [10]. Studies have suggested that serum cytokine levels, including IL-5 and IFN-γ, could be effective indicators for predicting the clinical efficacy and survival rates in patients with cancer undergoing anti-PD-1 blockade treatment, including those with lung cancer [11]. For chemoimmunotherapy settings, immunological and nutritional markers such as NLR and C-reactive protein-albumin ratio could also be useful for predicting the outcomes [12]. Also, high sPD-L1 concentration is a negative predictor of chemoimmunotherapy efficacy in patients with NSCLC [13]. In neoadjuvant settings, baseline NLR, PLR, monocyte-to-lymphocyte ratio (MLR), and systemic immune-inflammation index (SII) are associated with major pathological response (MPR) in NSCLC patients receiving neoadjuvant chemoimmunotherapy [14].

Here, we retrospectively analyzed the efficacy of PD-1 antibody therapy in locally advanced or metastatic NSCLC patients, and preliminarily explored the correlation between peripheral blood biomarkers and clinical efficacy thus enabling us to identify the population that would benefit most from PD-1-based therapy through a simple blood draw.

## Materials and methods

### Patient inclusion and exclusion criteria

This single-center, retrospective study was conducted at Nanjing Drum Tower Hospital, analyzing medical record data of 101 patients with stage IIIA–IV NSCLC treated with PD-1 antibody immunotherapy as a first-line or subsequent-line treatment, either as monotherapy or in combination with chemotherapy. The inclusion criteria were as follows: (1) An Eastern Cooperative Oncology Group performance status (ECOG PS) of 0–2, and receipt of ≥ 2 cycles of immunotherapy; (2) Diagnosis of stage IIIA–IV unresectable NSCLC; (3) Willingness to undergo blood collection before and after immunotherapy for serum cytokine testing and T cell phenotype analysis. The exclusion criteria were active autoimmune disease, severe infectious diseases, or systemic immunosuppression.

### Treatments and study assessments

Patients were administered albumin paclitaxel (260 mg/m^2^) or pemetrexed (500 mg/m^2^) in combination with carboplatin (AUC 5), lobaplatin (30 mg/m^2^), or cisplatin (75 mg/m^2^), and a PD-1 antibody (200 mg) every three weeks. For maintenance therapy, patients were treated with the PD-1 antibody, either with or without albumin paclitaxel or pemetrexed, according to pathology phenotype. After every two rounds of therapy, the Response Evaluation Criteria in Solid Tumors (RECIST, version 1.1) was employed to evaluate clinical efficacy. The criteria were as follows: complete response (CR): complete regression of the target lesions; partial response (PR): a reduction of more than 30% of the total target lesions; progressed disease (PD): a greater than 20% increase of the total target lesions; stable disease (SD): a reduction of less than 30% or an increase of less than 20% of the total target lesions; disease control rate (DCR): CR + PR + SD.

### Sample collection and flow cytometry

Blood samples were collected from 88 patients with NSCLC before and after two cycles of PD-1 antibody treatment concurrently with the first radiographic evaluation. Briefly, 10 mL of blood was obtained by venipuncture and collected into sterile tubes containing ethylenediaminetetraacetic acid. These samples were then transferred to the research laboratory within 2 h. Blood serum was collected and prepared for a mixed sample solution, as illustrated by the CBA assay kit for the detection of IL-2, IL-4, IL-5, IL-6, IL-10, IL-13, IFN-γ, and TNF-α (BD Biosciences, USA). Subsequently, peripheral blood mononuclear cells (PBMCs) were purified by Ficoll-Plaque (GE, Chicago, IL, USA) centrifugation and labeled for analysis with mouse monoclonal antibodies specific to CD3, CD4, CD8, CD279, CD45RO, CD62LC, D127, and CD25 (BioLegend, San Diego, CA, USA). The percentage of the relevant immune cell populations was quantified using FlowJo software (FlowJo LLC, Ashland, OR, USA).

### Statistical analysis

Statistical analysis was conducted using GraphPad Prism 9 software (GraphPad Software Inc., La Jolla, CA, USA) and the *R* programming language. The associations between cytokine levels, T-cell phenotype, and clinical response were analyzed using the paired *t-*test or the Wilcoxon test. Survival curves were calculated using the Kaplan–Meier analysis and compared using the unadjusted log-rank test. A *p*-value < 0.05 was considered statistically significant.

## Results

### Study population

**Fig. 1:**
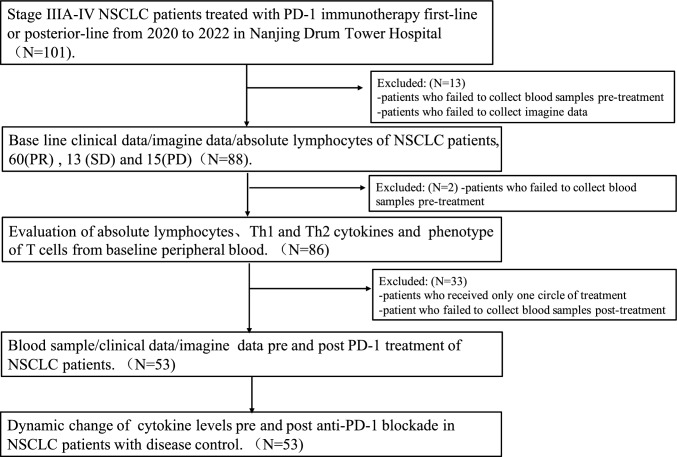
The overall flowchart of the study population and the method

Baseline clinical data, imaging data, and absolute lymphocytes of 88 patients (67 male and 21 female) with NSCLC were analyzed and approved by the Research Ethics Board of Drum Tower Hospital affiliated to Nanjing University. Absolute lymphocytes, Th1 and Th2 cytokines, and T cell phenotypes from the baseline peripheral blood of these patients were evaluated. Blood samples, clinical data, and imaging data before PD-1 treatment were also examined. The overall flowchart of the study population is shown in Fig. 1.

### Patient characteristics and clinical responses

In this retrospective study, 88 patients with stage IIIA–IV NSCLC who had undergone at least two cycles of PD-1 inhibitors between 2020 and 2022 were enrolled. Baseline patient characteristics were as follows: 56 patients had adenocarcinoma NSCLC, 26 had squamous NSCLC, and 6 had other types of NSCLC. In total, 60 patients (60/88, 68.18%) received PD-1 inhibitors as the first-line treatment. 79 patients received PD-1 inhibitors combined with chemotherapy (78/88, 89.77%) and 9 patients received PD-1 inhibitors alone (9/88, 10.23%). The median age of the patients was 61.5 years; most of them were male (67/88, 76.14%) and belonged to stage IV (65/88, 73.86%). Most patients had no sensitive driver gene mutations (67/88, 76.14%), whereas 17 had EGFR mutations (17/88, 19.31%), 2 had RET fusions (2/88, 2.27%), and 2 had KRAS G12C mutations (2/88, 2.27%). In total, 59 patients had an ECOG performance status of 0–1 (59/88, 67.05%), and 29 patients (29/88, 32.95%) had a poor performance status. All-stage immune-related adverse events occurred in 21.59% of the patients, but no severe adverse events were observed. According to the RECISTv1.1 criteria, 60 (68.18%) patients achieved a PR, 13 (14.77%) remained stable (SD), and 15 (17.05%) showed PD. The DCR was 82.95% (Table 1).

**Table 1:** Baseline patient characteristics and best clinical response

Characteristics		*N*	%
Age (YEARS)	Median < 70 > = 70	61.5 71 17	80.68 19.32
Gender	Male Female	67 21	76.14 23.86
Smoking status	Non-smoker Smoker	19 69	21.59 78.41
Histology	Adenocarcinoma Squamous Others	56 26 6	63.64 29.55 6.81
Line of treatment	< 2 > = 2	60 28	68.18 31.82
Treatment regimen	Albumin paclitaxel Pemetrexed Mono-immunotherapy	43 36 9	48.86 40.90 10.23
Clinical stage	III IV	23 65	26.14 73.86
Molecular status	EGFR mutation RET fusion KRAS G12C	17 2 2	19.31 2.27 2.27
PD-L1 status (TPS)	< 1%	1	1.14
	1–49%	7	7.95
	> 50% Unknown	7 73	7.95 82.95
ECOG performance status	0–1 > = 2	59 29	67.05 32.95
Immune adverse events (all grade)	Yes No	19 69	21.59 78.41
Best clinical response	PR SD PD	60 13 15	68.18 14.77 17.05

### Association between reduced lymphocyte absolute counts in peripheral blood and disease progression after anti-PD-1 blockade

**Fig. 2:**
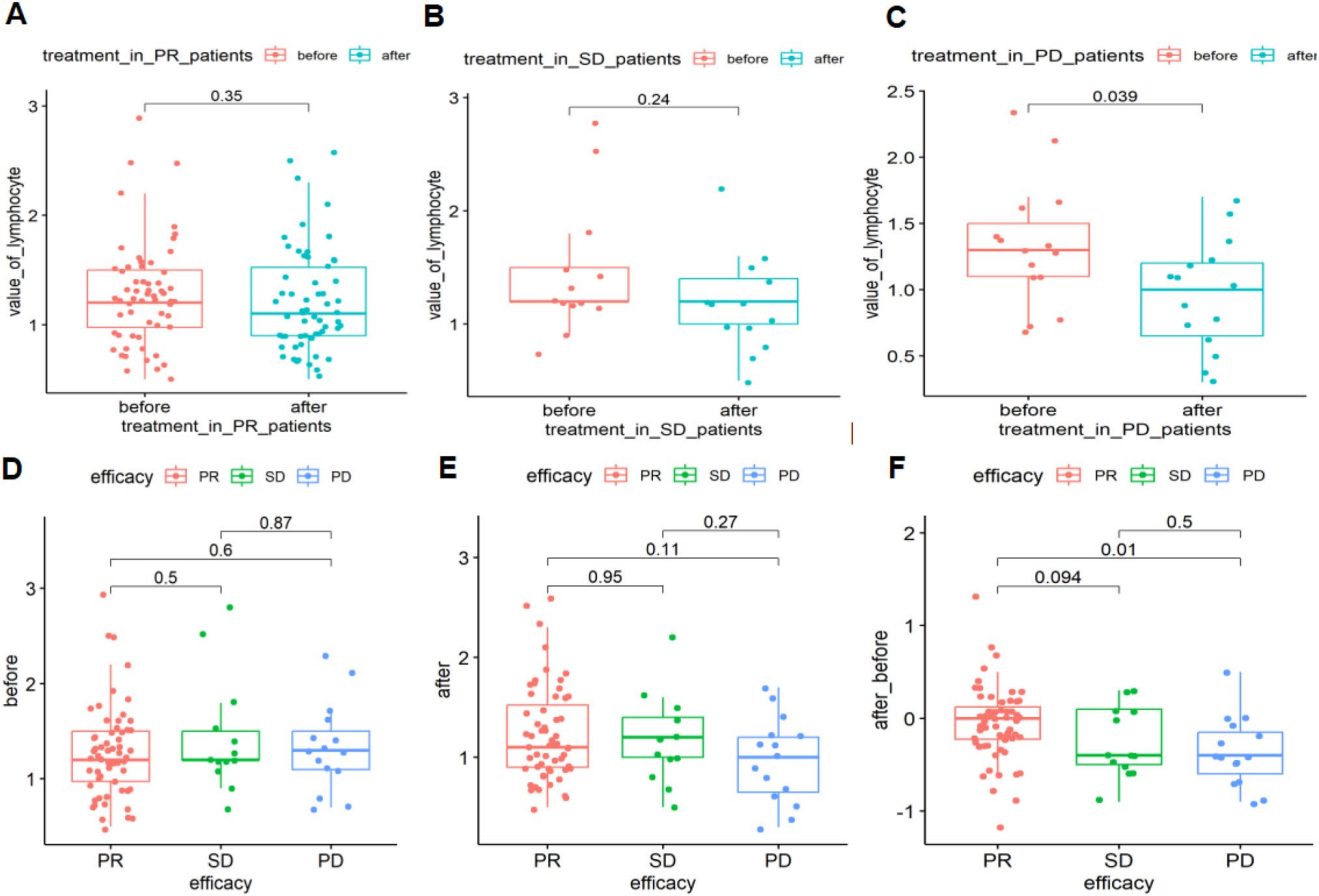
lymphocyte absolute counts in peripheral blood were associated with disease progression with anti-PD-1 blockade. **A** lymphocyte absolute counts in peripheral blood pre- and post-therapy in SD patients; **B** lymphocyte absolute counts in peripheral blood pre- and post-therapy in PD patients; **C** the dynamic change of absolute number of total lymphocytes in PR, SD, and PD patients. **D** lymphocyte absolute counts in peripheral blood pre-therapy in PR, SD, and PD patients; **E** lymphocyte absolute counts in peripheral blood post-therapy in PR, SD, and PD patients; **F** lymphocyte absolute counts in peripheral blood pre- and post-therapy in PR patients

We sought to determine whether the total lymphocyte absolute counts in peripheral blood were linked to the progression of the disease after anti-PD-1 blockade. Blood was collected from the patients at the beginning of treatment and during the initial clinical efficacy evaluation, typically two cycles after immunotherapy. There were no significant changes in lymphocyte counts before and after treatment between the PR (*P* = 0.35, *P* > 0.05) and SD (*P* = 0.24, *P* > 0.05) groups. However, a noteworthy decrease (*P* = 0.039, *P* < 0.05) in lymphocyte counts was observed in the PD group after treatment (PD, *n* = 15) (Fig. 2A–C). At the treatment baseline, there was no significant difference in the absolute lymphocyte counts between patients exhibiting PR (*n* = 60), SD (*n* = 13), and PD (*n* = 15) (*P* > 0.05: PR vs. SD; *P* = 0.5; PR vs. PD, *P* = 0.6; SD vs. PD, *P* = 0.87) (Fig. 2D). Similarly, no significant difference in the lymphocyte counts was observed between the PR, SD, and PD groups after treatment (*P* > 0.05: PR vs. SD, *P* = 0.95; PR vs. PD, *P* = 0.11; SD vs. PD, *P* = 0.27) (Fig. 2E). A dynamic reduction in total lymphocyte count was observed in the PD group, a trend not observed in the PR or SD group (*P* > 0.05: PR vs. SD, *P* = 0.094; *P* < 0.05, PR vs. PD, *P* = 0.01: *P* > 0.05, SD vs. PD, *P* = 0.5) (Fig. 2F). Overall, these results suggested that the reduction in the total lymphocyte counts in peripheral blood after PD-1 antibody-based therapy is associated with a poorer clinical response. This finding may also help identify patients who fail to benefit from immunotherapy, even before radiographic evaluation.

### Predictive value of serum cytokine levels for clinical response with anti-PD-1 blockade therapy in patients with NSCLC

**Fig. 3:**
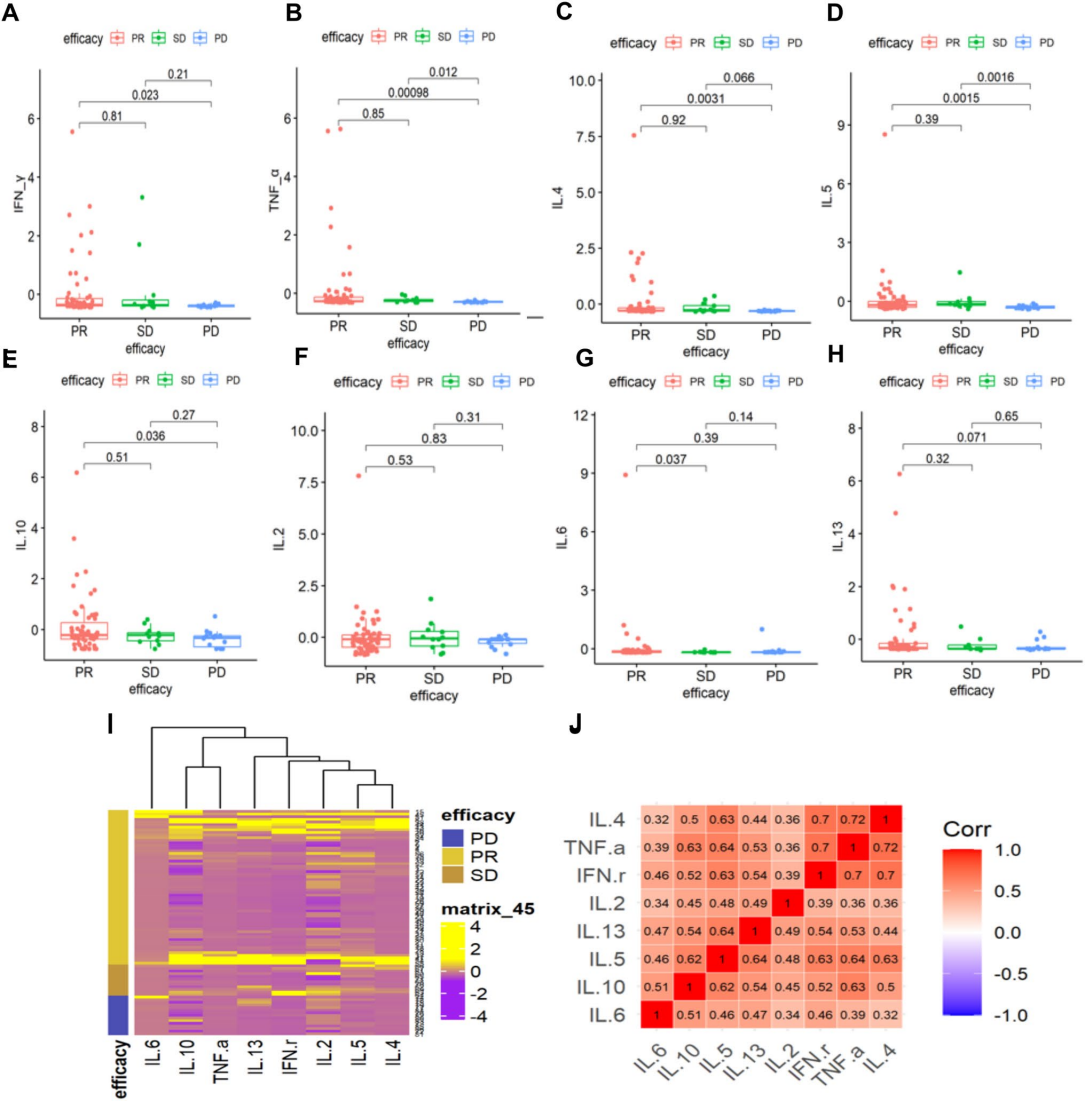
Predictive value of serum cytokine levels for clinical response to anti-PD-1 blockade therapy in patients with NSCLC **A** baseline IFN-γ levels; **B** TNF-α levels; **C** IL-4 levels; **D** IL-5 levels; **E** IL-10 levels; **F** IL-2 levels; **G** IL-6 levels; **H** IL-13 levels; and **I** cytokine heat map analysis of baseline cytokine levels in PR, SD, and PD patients; **J** cytokine correlation spearman analysis of IL-2, IL-4, IL-5, IL-6, IL-10, IL-13, IFN-γ, and TNF-α in blood serum prior to treatment

Blood serum samples were collected from 86 patients with late-stage NSCLC who received PD-1 or PD-L1 inhibitors. The levels of IL-2, IL-4, IL-5, IL-6, IL-10, IL-13, IFN-γ, and TNF-α in the blood serum were tested and analyzed before treatment. In the group with superior clinical response, defined by PR (*n* = 60), higher levels of IFN-*γ* (*P* = 0.023, *P* < 0.05) (Fig. 3A), TNF-*α* (*P* = 0.00098, *P* < 0.005) (Fig. 3B), IL-4 (*P* = 0.0031, *P* < 0.005) (Fig. 3C), IL-5 (*P* = 0.0015, *P* < 0.005) (Fig. 3D), and IL-10 (*P* = 0.036, *P* < 0.05) (Fig. 3E) were detected in peripheral blood prior to the treatment compared to the treatment ineffective group (PD, *n* = 15). However, the serum levels of IL-2 (*P* = 0.83, *P* > 0.05) (Fig. 3F), IL-6 (*P* = 0.39, *P* > 0.05) (Fig. 3G), and IL-13 (*P* = 0.071, *P* > 0.05) (Fig. 3H) had no correlation with the clinical response to PD-1 blockade.

Overall, these results suggested that high levels of the systemic Th1 cytokines, such as IFN-*γ* and TNF-*α*, and Th2 cytokines such as IL-5, IL-4, and IL-10 preexisting in patients with NSCLC could be good indicators of objective clinical responses to PD-1 blockade. The baseline cytokine secretion status of patients in different therapeutic groups is intuitively shown in the heat map in Fig. 3I. In addition, we found a certain correlation between different cytokines through Spearman correlation analysis. There was a strong correlation between IFN-*γ*, TNF-*α*, IL-5, IL-4, and IL-10 compared to other cytokines (Fig. 3J).

### Predictive value of serum cytokine levels for progression-free survival after anti-PD-1 blockade therapy in patients with NSCLC

**Fig. 4:**
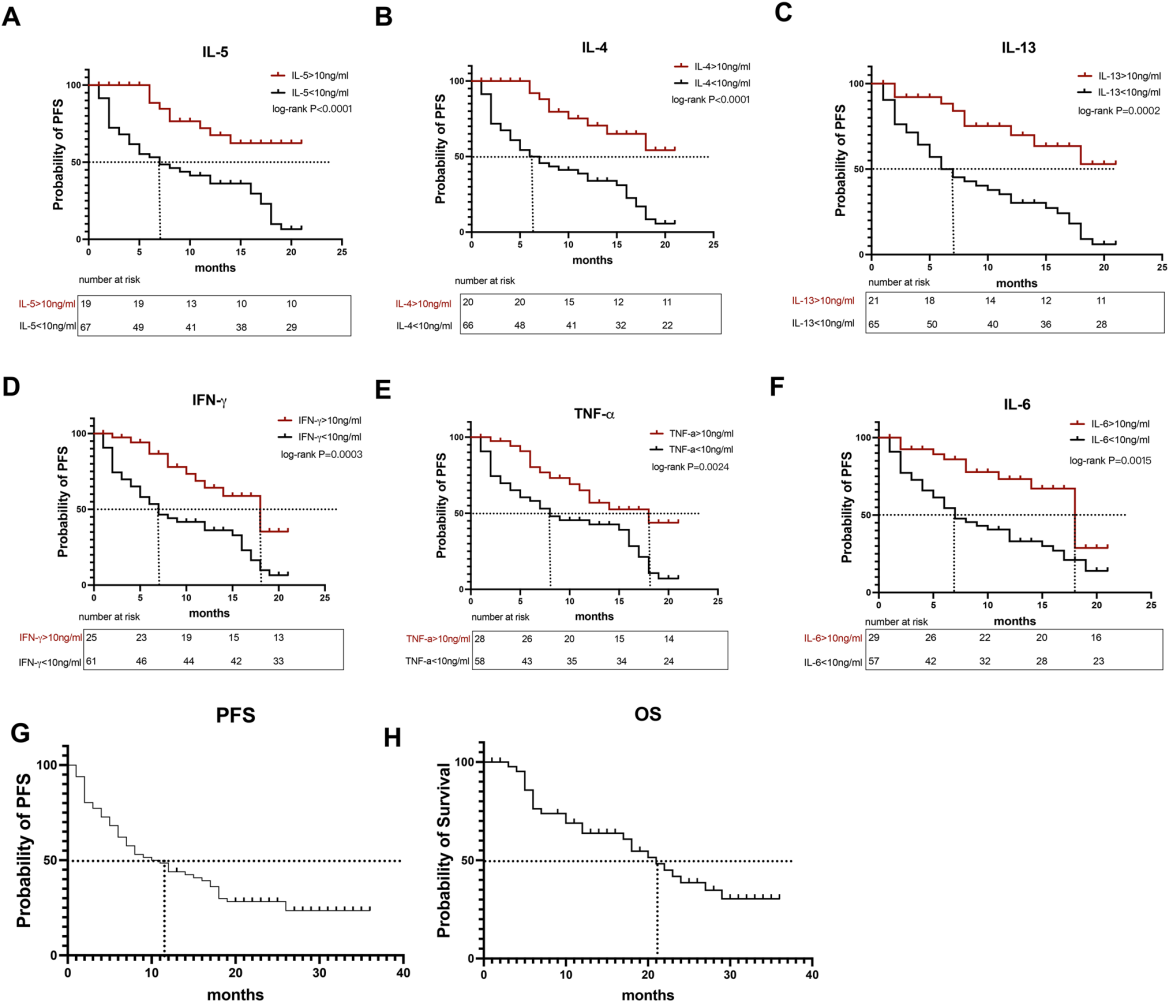
Predictive value of serum cytokine levels of progression-free survival with anti-PD-1 blockade therapy in NSCLC patients **A** predictive value of IL-5 levels of progression-free survival of NSCLC patients with anti-PD-1 blockade therapy; **B** predictive value of IL-4 levels of progression-free survival of NSCLC patients with anti-PD-1 blockade therapy; **C** predictive value of IL-13 levels of progression-free survival of NSCLC patients with anti-PD-1 blockade therapy; **D** predictive value of IFN-γ levels of progression-free survival of NSCLC patients with anti-PD-1 blockade therapy; **E** predictive value of TNF-α levels of progression-free survival of NSCLC patients with anti-PD-1 blockade therapy; **F** predictive value of IL-6 levels of progression-free survival of NSCLC patients with anti-PD-1 blockade therapy; **G** the Kaplan–Meier curves for PFS in the overall cohort; **H** he Kaplan–Meier curves for OS in the overall cohort

As shown in Fig. 4, the progression-free survival (PFS) of patients with late-stage NSCLC who received PD-1 inhibitors was analyzed based on multiple serum cytokine levels collected before immunotherapy. Higher baseline cytokine levels (> 10 ng/mL) were found to be positive prognostic factors for patients with advanced NSCLC treated with immunotherapy. Patients with high IL-5 levels (> 10 ng/mL) had superior median progression-free survival (mPFS) compared to those with low IL-5 levels (< 10 ng/mL) (undefined vs. 7 months, HR 0.237, 95%CI 0.14–0.42, *P* < 0.0001) (Fig. 4A). Patients with high IL-4 levels (> 10 ng/mL) had superior mPFS compared to those with low IL-4 levels (< 10 ng/mL) (undefined vs. 6.5 months, HR 0.225, 95%CI 0.13–0.39, *P* < 0.0001) (Fig. 4B). Similarly, patients with high IL-13 levels (> 10 ng/mL) had superior mPFS compared to those with low IL-13 levels (< 10 ng/mL) (undefined vs. 6.5 months, HR 0.270, 95%CI 0.15–0.47, *P* = 0.0002) (Fig. 4C). Patients with high IFN-*γ* levels (> 10 ng/mL) had superior mPFS compared to those with low IFN-*γ* levels (< 10 ng/mL) (18 vs. 7 months, HR 0.333, 95%CI 0.19–0.58, *P* = 0.0003) (Fig. 4D). Similarly, patients with high TNF-*α* levels (> 10 ng/mL) had superior mPFS compared to those with low TNF-*α* levels (< 10 ng/mL) (18 vs. 8 months, HR 0.41, 95%CI 0. 23–0.73, *P* = 0.0024) (Fig. 4E). Patients with high IL-6 levels (> 10 ng/mL) had superior mPFS compared to those with low IL-6 levels (< 10 ng/mL) (18 vs. 7 months, HR 0.38, 95%CI 0.22–0.68, *P* = 0.0015) (Fig. 4F). Despite the prevalence of low IL-2 and IL-10 levels in most patients with late-stage NSCLC prior to receiving PD-1 inhibitors, the cutoff value was hard to determine and was, thus, not taken into account in the analysis. The mPFS was 10.5 months and the mOS was 21 months in the overall cohort. Also, The Kaplan–Meier curves for PFS and OS in the overall cohort were shown in Figs. 4G and H.

In summary, the survival analysis revealed that patients with higher baseline cytokine levels, specifically IL-5, IL-4, IL-13, IFN-*γ*, TNF-*α*, and IL-6, had a longer PFS upon receiving immunotherapy.

### Dynamic changes of cytokine levels were not associated with disease control related with anti-PD-1 blockade in patients with NSCLC

**Fig. 5:**
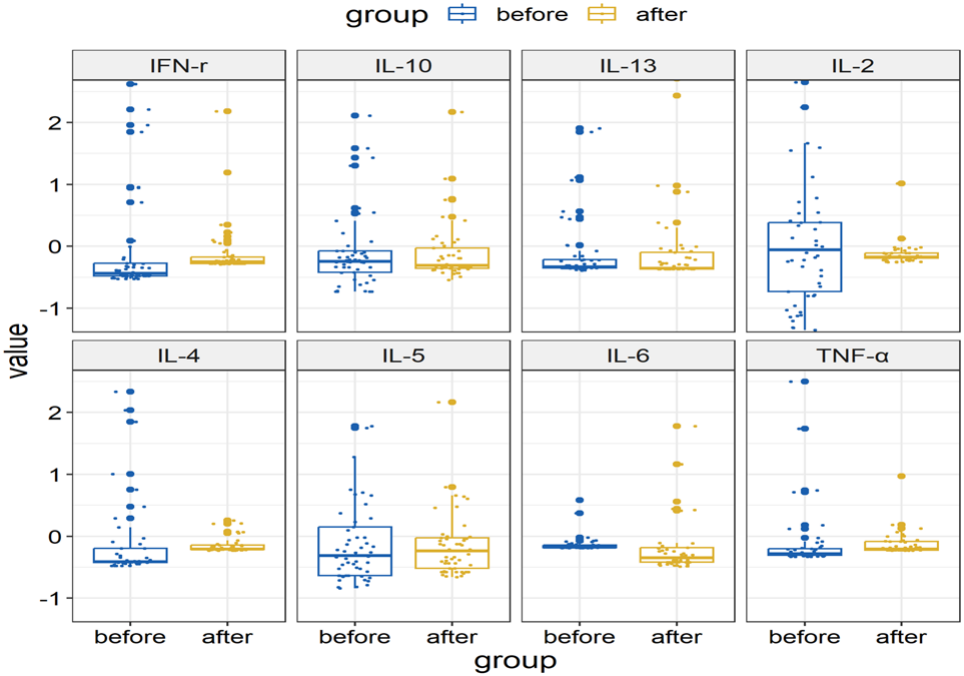
Dynamic cytokine levels pre- and post-anti-PD-1 blockade in NSCLC patients with disease control **A** the dynamic IFN-γ levels pre- and post-anti-PD-1 blockade in NSCLC patients with disease control; **B** the dynamic TNF-α levels pre- and post-anti-PD-1 blockade in NSCLC patients with disease control; **C** the dynamic IL-5 levels pre- and post-anti-PD-1 blockade in NSCLC patients with disease control; **D** the dynamic IL-2 levels pre- and post-anti-PD-1 blockade in NSCLC patients with disease control; **E** the dynamic IL-6 levels pre- and post-anti-PD-1 blockade in NSCLC patients with disease control; **F** the dynamic IL-10 levels pre- and post-anti-PD-1 blockade in NSCLC patients with disease control; **G** the dynamic IL-4 levels pre- and post-anti-PD-1 blockade in NSCLC patients with disease control; **H** the dynamic IL-13 levels pre- and post-anti-PD-1 blockade in NSCLC patients with disease control

To investigate the effects of PD-1 blockade on immune responses during cancer immunotherapy and to identify the population that could benefit from immunotherapy, we analyzed Th1/Th2 cytokines from the peripheral blood of 53 patients with NSCLC at baseline and at the first treatment evaluation, usually after two cycles of treatment. Serum samples were collected from a cohort of patients who showed disease control, classified as PR or SD. Th1/Th2 cytokines including IL-2, IL-4, IL-5, IL-6, IL-10, IL-13, IFN-*γ*, and TNF-*α* were evaluated. In patients showing PR or SD, neither of the Th1/Th2 cytokines showed significant upregulation or downregulation after treatment. As illustrated in Fig. 5, the serum levels of IFN-*γ* (P = 0.576, *P* > 0.05; Fig. 5A), TNF-*α* (*P* = 0.853, *P* > 0.05; Fig. 5B), IL-5 (*P* = 0.220, *P* > 0.05; Fig. 5C), IL-2 (*P* = 0.300, *P* > 0.05; Fig. 5D), IL-6 (*P* = 0.471, *P* > 0.05; Fig. 5E), IL-10 (*P* = 0.276, *P* > 0.05; Fig. 5F), IL-4 (*P* = 0.892, *P* > 0.05, Fig. 5G), and IL-13 (*P* = 0.218, *P* > 0.05, Fig. 5H) showed insignificant changes after treatment in patients who benefitted from treatment.

### Predictive value of T cell phenotypes and clinical response following anti-PD-1 blockade

Since the absolute counts of lymphocytes were correlated with clinical response to immunotherapy, we sought to determine whether a specific T cell phenotype contributes to its efficacy. To identify the correlation between existing T cell phenotypes in the peripheral blood of patients with NSCLC and their clinical response during cancer immunotherapy with PD-1/PD-L1 blockade, we conducted flow cytometry analyses of CD4^+^ and CD8^+^ T cells derived from the PBMCs of patients with NSCLC at baseline. PBMCs were isolated and stained with flow cytometry antibodies including inhibitory markers PD-1, regulatory T cell markers CD25 and CD127, and memory and effector markers CD45RO and CD62L. Fresh or frozen samples were gated on live CD3^+^ CD4^+^ T cells or CD3^+^ CD8^+^ T cells, followed by analysis of specific phenotype markers. Most samples were taken both before and after PD-1 blockade treatment.

**Fig. 6:**
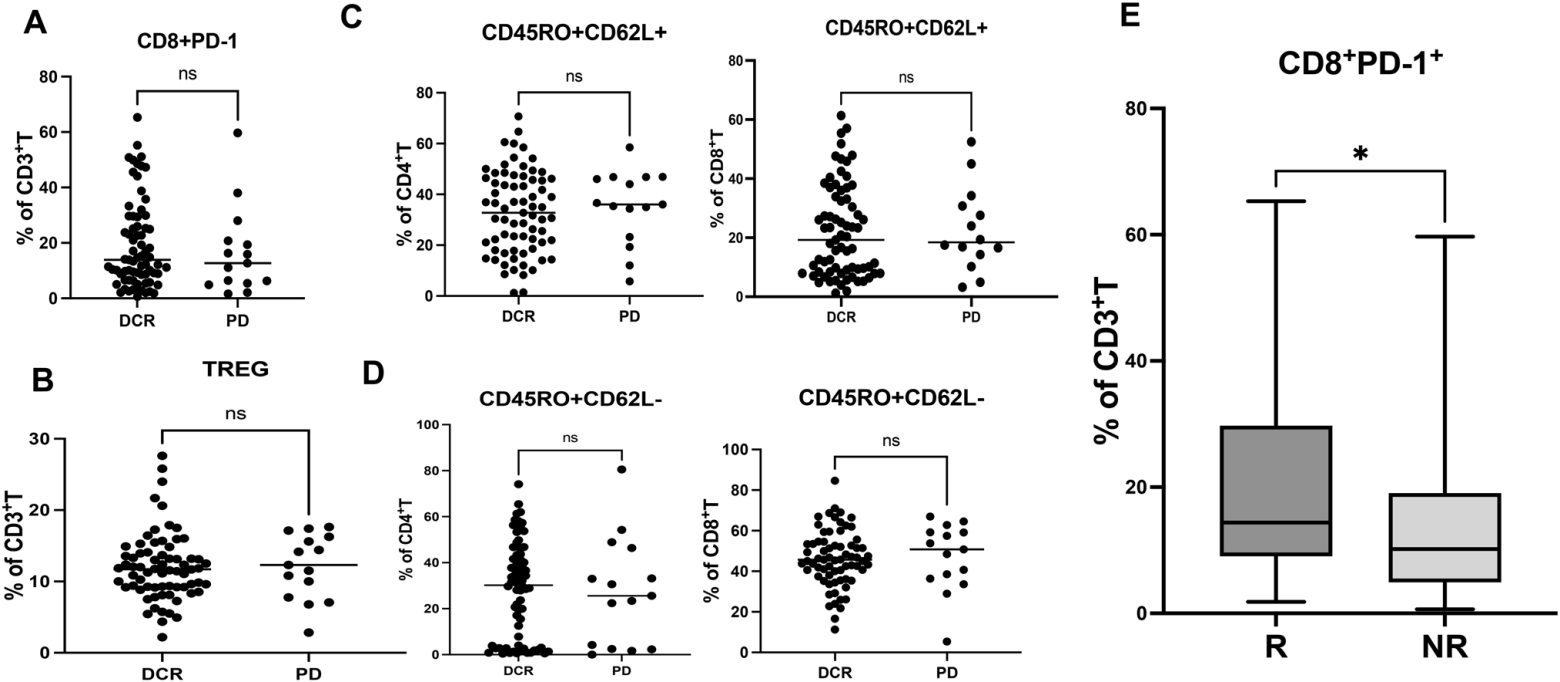
Predictive value of T cells phenotype and clinical response of anti-PD-1 blockade **A** the percentage of CD8^+^PD-1^+^T cells in PBMCs at the baseline from DCR patients and PD patients; **B** the percentage of Treg cells in PBMCs at the baseline from DCR patients and PD patients; **C** the percentage of CD4^+^CD45RO^+^CD62L^+^T cells and CD8^+^CD45RO^+^CD62L^+^T cells in PBMCs at the baseline from DCR patients and PD patients; **D** the percentage of CD4^+^CD45RO^+^CD62L^−^T cells and CD8^+^CD45RO^+^CD62L^−^T cells in PBMCs at the baseline from DCR patients and PD patients; **E** the percentage of CD8^+^PD-1^+^T cells in PBMCs at the baseline from responding patients and non-responding patients

We then assessed whether baseline phenotypic profiles of T cells differed between responding (DCR) and non-responding (PD) patients. As summarized in Fig. 6, most of the markers on CD4^+^ T cells or CD8^+^ T cells did not significantly differ between the two groups. Specifically, there were no differences in PD-1 expression on CD8^+^ T cells between the DCR and PD patients (*P* = 0.479, *P* > 0.05; Fig. 6A). Likewise, the percentage of Treg cells did not significantly differ between the two groups (*P* = 0.634, *P* > 0.05; Fig. 6B). Similarly, there was no difference in the distribution of the central memory (CD45^+^CD62L^+^) and effector memory (CD45^+^CD62L^−^) markers on both CD4^+^ T cells and CD8^+^ T cells between the DCR and PD patients (*P* > 0.05, Fig. 6C and D). However, we found that although there were no differences in PD-1 expression on CD8^+^ T cells between the DCR and PD patients, this expression was associated with the clinical response between responding patients (PR) and non-responding patients (SD + PD) (*P* = 0.042, *P* < 0.05; Fig. 6E), indicating that responding patients may have different T cell phenotypes that warrant further exploration. The lack of significant results in other aspects indicates the challenges of using T cell phenotypes to identify patients who would clinically benefit from PD-1/PD-L1 inhibitors in the context of NSCLC.

## Discussion

PD-L1 is currently a known prognostic indicator for the efficacy of immunotherapy in NSCLC. However, even in patients who are PD-L1 negative, a combination of chemotherapy and immunotherapy has been found to be effective [15]. This approach has led to an increased response rate and a higher overall survival (OS) rate. However, some patients still exhibit primary resistance to immune checkpoint blockade therapy, and some acquire resistance during the immunotherapy process. The exact mechanisms underlying the resistance to PD-1 blockade therapy are not yet well understood; however, they likely involve multiple factors including an abnormal gut microbiome composition [16], parallel immune inhibitory pathways such as Tim-3 or Lag-3 [17], and a loss of antigen-presentation capacity. To address this concern, research has been conducted to identify peripheral blood biomarkers that can signal a response to PD-1 blockade in patients with NSCLC.

Through this study, we suggested that the reduction of lymphocyte absolute counts in patients with PD may also help us to identify patients who may not benefit from immunotherapy even before image evaluations. Our analysis also uncovered that baseline blood serum cytokine levels could predict the effectiveness of anti-PD-1 blockade therapy in patients with NSCLC. We found that elevated levels of Th1 cytokines such as IFN-γ and TNF-α, as well as Th2 cytokines such as IL-5, IL-4, and IL-10, already existing in patients with NSCLC, could reliably predict clinical responses to PD-1 blockade therapies. Furthermore, our results showed a positive correlation between higher baseline cytokine levels (> 10 ng/mL) and improved PFS in patients with advanced NSCLC undergoing immunotherapy. The role of IFNs as potent immunomodulators is important, guiding the actions on both innate and adaptive lymphocytes, augmenting natural killer cell cytotoxicity, and augmenting dendritic cell function—all of which are essential for the initiation of adaptive immune responses that inhibit tumor development [18].

The recruitment and activation of neutrophils, macrophages, and lymphocytes at sites of damage, infection, and tumor development [19] is a result of TNF-α and other proinflammatory factors. This is in agreement with the findings that higher IFN-*γ* and TNF-*α* cytokine levels at the time of diagnosis and three months after treatment initiation are linked to a better response to immunotherapy and a longer OS in NSCLC [20]. Our results indicated that a preexisting Th1 cytokine response may help trigger a robust and long-lasting immune response after PD-1 antibody blockade. While tumor immunity is mainly governed by Th1-mediated cellular responses, we observed that preexisting higher levels of IL-5, IL-4, and IL-10 were also correlated with better clinical responses. Specifically, higher levels of IL-5 were associated with a significant prognostic effect. This may be attributed to the role of T helper-2 (Th2) lymphocytes and group 2 innate lymphoid cells (ILC2) in antibody production. These cells can boost the humoral immune response through the differentiation and growth of B cells, thereby promoting antibody response [21].

The immune-inflammatory state of the patients may be indicative of their heightened sensitivity to immunotherapy, rather than an inadequate immune response. In patients with NSCLC who undergo anti-PD-1 treatment, the dynamic alterations of cytokine levels could not be used to predict clinical responses, even among those showing a stable or clinical response. We found that serum cytokines are unsuitable for disease monitoring, because the dynamic changes of cytokine levels may be affected by multiple factors including different treatments such as chemotherapy, the immune status of the patient, or treatment-related side effects. This aligns with previous findings that baseline serum cytokine levels could be used to predict the benefits of immunotherapy, but they are unsuitable for longitudinal disease monitoring [22].

Accumulating evidence has suggested that CD8^+^ T cells in the tumor microenvironment and systemic CD4^+^ T-cell immunity have a significant role in maintaining antitumor responses. Previous research has shown that central memory CD4^+^ T cells in peripheral blood can be used as predictors of PD-1 blockade therapy in patients with malignant melanoma [23]. Previous studies have shown that CD70, the ligand for CD27, is essential for the successful priming of CD8^+^ T cells and successful antitumor immunity [24–30]. However, in our research, the majority of markers on CD4^+^ T cells or CD8^+^ T cells did not show any significant differences between responders and non-responders. In our previous study, we noticed a correlation between a greater proportion of CD45RO^+^CD62L (central memory phenotype) to CD45RO^+^CD62L (effecter memory phenotype) on CD4^+^T cells and a more favorable clinical outcome of PD-1 blockade in patients with lung cancer, although the results were not statistically significant, possibly owing to the limited sample size. Even with an increased sample size in our recent study, these markers did not yield significant insights in predicting the PD-1 blockade outcomes in patients with NSCLC.

Previous research has suggested that certain biomarkers in the peripheral blood may be possible indicators of the effectiveness of PD-1 blockade immunotherapy in lung cancer. Of these, serum inflammation and nutritional markers are simple to assess in clinical settings and can be easily incorporated into clinical practice. Research suggests that pretreatment levels of AEC, AMC, ALB, NLR, and PLR were independent positive predictors of PD-1 inhibitors in patients with advanced NSCLC [9]. In another study, patients with NLR values < 5, LDH levels < 240 U/L, or PNI ≥ 45 had significantly better outcomes. Multivariable analysis revealed that these parameters had an independent correlation with both enhanced PFS and OS [8]. Some studies have indicated that dynamic changes of serum makers are associated with clinical outcomes. Patients whose NLR decreased six weeks after treatment tended to have a longer PFS, and similar results were found in repeated measurements [31].

However, these studies are all retrospective and have a relatively small sample size. The predictive value of these blood serum markers on PFS or OS requires further validation by randomized studies with larger sample sizes and control groups. Moreover, there is an inconsistency in the cutoff values for these immune-inflammatory nutritional parameters across published studies, rendering the standards of evaluation challenging.

No significant treatment- or response-associated phenotypic differences were observed in bulk CD8 + T cells in several studies, which is in line with our results. Investigations into PD-1 expression on peripheral blood cells have demonstrated that it can enrich tumor-reactive cells in certain contexts [32–34]. Additionally, research has indicated that the proliferation of PD-1^+^ CD8^+^ T cells after PD-1 targeted therapy may be linked to clinical outcomes [35]. To further explore PD-L1 expression on peripheral blood cells, an exploratory study was conducted involving 32 patients with NSCLC undergoing PD-L1/PD-1 blockade therapies. A marked disparity in the proportion of PD-L1^+^ CD11b^+^ myeloid cells between objective responders and non-responders was observed; those with a PD-L1^+^ CD11b^+^ cell proportion above 30% initially demonstrated a response rate of 50% [36]. Moreover, the expression of CD3^+^CD8^+^PD-1^+^ cells in peripheral blood, when compared between DCR and PD patients, revealed a greater response to PD-1/PD-L1 blockade, indicating that the percentage of PD-1^+^ cell populations among peripheral blood T cells could distinguish between objective responders and non-responders.

Given the minimal differences observed in the study, future research should focus on more specific cell populations, such as neoantigen-specific T cells. Studies showed that the diversity of tumor-antigen-related immune cells may also play a predictive role, in addition to the prevalence of PD-1^+^ T cells. Studies have revealed that the diversity of peripheral PD-1^+^CD8^+^ TCR and the presence of neoantigen-specific CD8^+^ T cells could predict the clinical benefits of anti-PD-1/PD-L1 therapy. Immunotherapy has caused a transformation in the TCR repertoire, with early changes in TCR clonality correlating with the immune response, thus influencing the clinical outcomes of anti-PD-1/PD-L1 treatment in NSCLC [33, 37–40].

Our research has several restrictions. Firstly, it was a retrospective single-center study and the number of participants was limited, which underscores the need for paired pre- and post-treatment patient samples to better understand the dynamic changes. Secondly, we included all patients treated with PD-1 antibodies in combination with chemotherapy but did not establish a control group of patients who received only chemotherapy. Consequently, changes in cytokine levels or immune cell phenotypes might be influenced by the chemotherapeutic agents and are not only attributable to immunotherapy. Thirdly, the cutoff values of these parameters were not clearly defined; using different cutoff values could significantly influence the outcomes. In this study, 31.82% of patients were administered PD-1 inhibitors as their second or post-subsequent line of treatment. The baseline blood markers may have been affected by previous treatments including chemotherapy and other agents.

Despite these limitations, to the best of our knowledge, our study is the first to identify pretreatment Th1/Th2 cytokine levels in the peripheral blood of patients with NSCLC as potential indicators of response efficacy of and survival benefits from anti-PD-1/PD-L1 immunotherapy.

In conclusion, our study suggests that pretreatment serum cytokine levels could predict the clinical efficacy as well as PFS in patients with NSCLC undergoing anti-PD-1 blockade therapy. Patients showing reduced total lymphocyte counts after immunotherapy might experience poorer clinical outcomes. Moreover, a higher CD3^+^CD8^+^PD-1^+^T cell count in peripheral blood prior to treatment correlated with a more favorable objective clinical response. These biomarkers could help clinicians identify subpopulations that are more likely to benefit from anti-PD-1/PD-L1 immunotherapy therapy. The significance of these biomarkers warrants detailed investigation through extensive prospective studies in the future.

## Data Availability

Due to local restrictions on the sharing of biological data, the data generated/analyzed from this study is available via corresponding author upon request.
